# Blood pressure at age 60–65 versus age 70–75 and vascular dementia: a population based observational study

**DOI:** 10.1186/s12877-017-0649-3

**Published:** 2017-10-27

**Authors:** Mingkai Peng, Guanmin Chen, Karen L. Tang, Hude Quan, Eric E. Smith, Peter Faris, Vladimir Hachinski, Norm R. C. Campbell

**Affiliations:** 1 Department of Community Health Sciences, University of Calgary, Calgary, T2N 1N4 Canada; 2 Alberta Health Services, Calgary, T2N 4L7 Canada; 3 Cumming School of Medicine, University of Calgary, Calgary, T2N 1N4 Canada; 4 Department of Clinical Neurosciences, University of Calgary, Calgary, T2N 1N4 Canada; 5 Department of Clinical Neurological Sciences, London Health Sciences Centre, Western University, London, ON N6A 5A5 Canada; 6 Department of Medicine, Physiology and Pharmacology and Community Health Sciences, O’Brien Institute for Public Health and Libin Cardiovascular Institute of Alberta, University of Calgary, Calgary, AB T2N 1N4 Canada

**Keywords:** Population based observational study, Blood pressure, Vascular dementia, Pulse pressure

## Abstract

**Background:**

Vascular dementia (VaD) is the second most common form of dementia. However, there were mixed evidences about the association between blood pressure (BP) and risk of VaD in midlife and late life and limited evidence on the association between pulse pressure and VaD.

**Methods:**

This is a population-based observational study. 265,897 individuals with at least one BP measurement between the ages of 60 to 65 years and 211,116 individuals with at least one BP measurement between the ages of 70 to 75 years were extracted from The Health Improvement Network in United Kingdom. Blood pressures were categorized into four groups: normal, prehypertension, stage 1 hypertension, and stage 2 hypertension. Cases of VaD were identified from the recorded clinical diagnoses. Multivariable survival analysis was used to adjust other confounders and competing risk of death. All the analysis were stratified based on antihypertensive drug use status. Multiple imputation was used to fill in missing values.

**Results:**

After accounting for the competing risk of death and adjustment for potential confounders, there was an association between higher BP levels in the age 60–65 cohort with the risk of developing VaD (hazard ratio [HR] 1.53 (95% confidence interval: 1.04, 2.25) for prehypertension, 1.90 (1.30, 2.78) for stage 1 hypertension, and 2.19 (1.48, 3.26) for stage 2 hypertension) in the untreated group. There was no statistically significant association between BP levels and VaD in the treated group in the age 60–65 cohort and age 70–75 cohort. Analysis on Pulse Pressure (PP) stratified by blood pressure level showed that PP was not independently associated with VaD.

**Conclusion:**

High BP between the ages of 60 to 65 years is a significant risk for VaD in late midlife. Greater efforts should be placed on early diagnosis of hypertension and tight control of BP for hypertensive patients for the prevention of VaD.

**Electronic supplementary material:**

The online version of this article (10.1186/s12877-017-0649-3) contains supplementary material, which is available to authorized users.

## Background

Hypertension and dementia are common disorders in the elderly. Among people aged 60 years and over, the prevalence is estimated to be around 5 to 7% for dementia and over 50% for hypertension [1, 2]. Vascular dementia (VaD) caused by a variety of vascular diseases is one of the most common forms of dementia [3]. There are several different sets of clinical diagnostic criteria for VaD, though each set tends to consist of a combination of history of ischemic stroke, temporal relationship between onset of dementia and stroke, and/or evidence of cerebrovascular disease. High blood pressure (BP), or hypertension, is the leading risk factor for stroke and many other vascular diseases [4]. Thus, hypertension likely plays an important role in the development of VaD, though this association remains unclear [5].

The association between BP and dementia may be age dependent, [6, 7] with high BP at midlife (age 40–64 years) being associated with an increased risk for VaD [5, 8]. In contrast, there is no consensus on the association between BP and dementia in those aged 75 and above. A history of hypertension at age 75 or over is not consistently a risk factor for VaD, with some studies even reporting an inverse association between level of blood pressure and risk of VaD [9, 10]. Previous studies have been focused on the association of systolic and diastolic BP with dementia. Pulse pressure (PP) is the difference between the systolic and diastolic pressure readings, and is a measure of arterial stiffness. The pulsatile component of BP and high PP has been shown to increase the risk of cardiovascular disease and total mortality [11]. The association of PP with dementia remains unclear.

Electronic medical records (EMR) contain rich clinical information, in which more than 50% of patients have at least 2 blood pressure measurements [12]. EMR also provides a population-based sample with the ability to follow patients for long periods of time. The objective of this study was to investigate the association of BP and PP levels in late midlife and late-life with the risk of vascular dementia using United Kingdom (UK) primary care data. We developed two separate study cohorts (aged 60–65 and aged 70–75) to elucidate whether there is an age-dependent relationship of blood pressure with VaD.

## Methods

### Data source

This study was conducted using The Health Improvement Network (THIN) that is one of the largest source of continuous primary care data in United Kingdom (UK). Prospective data collection in THIN started in 2002 with data dating back to the early 1980s. THIN received anonymized patient data from general practice computer systems used for patient management and research purposes. It is a primary care EMR database with the patient information recorded during routine physician visits, such as diagnoses, prescriptions, physiological measurements, diagnostic tests, lifestyle information, and referrals to secondary care. THIN holds anonymized longitudinal medical records for over 11.1 million individuals with more than 3.7 million who remain actively registered. THIN is demographically representative of UK [13]. The Read clinical classification, developed in the UK for primary care, was used in THIN to code clinical information [14].

### Study population

We conducted a retrospective population-based cohort study. Two independent study cohorts were created. The first cohort included individuals with at least one blood pressure measurement between the ages of 60 to 65, and the second cohort included individuals with at least one blood pressure measurement between the ages of 70 and 75. We used the date of last blood pressure measurement within 60 to 65 or 70 to 75 as the index date of follow-up. To ensure an adequate period of follow-up, we only included individuals with an index date between the years 1989 and 2004 and who have registered in THIN for at least 2 years before the index date. All individuals were followed until they experienced the any one of the following: diagnosis of VaD, died, left the THIN database, or the follow-up ended (14 May 2012). Anyone with a diagnosis of VaD before the index date was excluded.

### Blood pressure categories

We extracted all the blood pressure values measured within 2 years prior to the index date and calculated the average systolic (SBP) and diastolic blood pressure (DBP). BP (measured in mmHg) were classified into 4 categories: normal: SBP < 120 and DBP < 80; prehypertension: 120 ≤ SBP < 140 or 80 ≤ DBP < 90; stage 1 hypertension: 140 ≤ SBP < 160 or 90 ≤ DBP < 100; stage 2 hypertension: SBP ≥ 160 or DBP ≥ 100 according to the criteria of the seventh report of Joint National committee on the Prevention, Detection, Evaluation and Treatment of High Blood Pressure (JNC-7) [15]. Individuals were classified into the higher category if there was a difference in categories based on diastolic and systolic blood pressure. We categorized PP (measured in mmHg) into 4 categories: PP ≤ 50, 50 < PP ≤ 60, 60 < PP ≤ 70, and 70 < PP.

### Ascertainment of vascular dementia

We ascertained the cases of VaD using the diagnosis codes E004 - arteriosclerotic dementia/multi-infarct dementia and Eu01 - vascular dementia/arteriosclerotic dementia. The index date of VaD was the earliest diagnosis date recorded in the THIN. Diagnosis information in THIN are generally well recorded and have been successfully used in several studies on dementia [16, 17]. According to the diagnosis guideline, the general practitioners made a diagnosis of dementia through the following steps: patient history review, cognitive impairment assessment through a specific test (e.g mini-mental state examination test), referral to a specialist, and/or assessment based on neuroimaging techniques [18]. The coding of dementia in EMR data shows good agreement with physician diagnosis and hospital admission data [19, 20].

### Other risk factors

Individuals with the diagnosis of diabetes, stroke, and depression prior to the index date were identified using the Read codes listed in Quality and Outcome Framework- National Health Science (QOF-NHS). History of head injury and Parkinson’s disease was identified based on Read codes found in a text search in the Read code dictionary [21]. All codes used in this study are listed in Additional file 1: Table S1. We also extracted smoking status of individuals using the following three classifications: never smoked, former smoker, and current smoker. Body mass index (BMI) was calculated based on the recorded weight and height. We used the median height recorded after the age of 25 and the most recent weight recorded within 5 years prior to the index date of follow-up. BMI were classified into three categories: normal (<25), overweight (25 ≤ and <30), and obesity (≥ 30).

We extracted all the antihypertensive drugs information (See Additional file 1: Table S2 for drug list) 1 year before the study index date. Anyone who had at least one antihypertensive drug prescription were assigned as the treated group.

### Statistical analysis

Descriptive statistics were used to compare the demographics and clinical conditions for patients with different blood pressure levels. We explored the association of blood pressure and vascular dementia by estimating age and sex standardized incidence rates of 13 SBP categories and 7 DBP categories. Blood pressure was categorized for every 5 mmHg interval from 115 to 170 mmHg for SBP and from 70 to 95 mmHg for DBP. We categorized individuals with SBP/DBP below or above the ranges as the first and last categories (for example, for SBP, < 115 or >170 mmHg). The mean blood pressure value for each category was calculated. The cumulative incidence of vascular dementia was estimated using the product-limit life-table method, accounting for the competing risk of death [22]. Proportional subdistribution hazard model was used to estimate the association of blood pressure levels and PP with risk of VaD, while treating death as a competing risk [23]. Stratified analysis on PP at each BP level was conducted using the proportional hazard model. Adjustment for potential confounders including age, sex, smoking status, BMI categories, and history of diabetes, stroke, depression, and Parkinson’s disease was performed in all models. All of the analyses were stratified by drug use status (treated and untreated).

The variables of smoking status and BMI had missing values. We approached this issue using two different methods. First, models were developed using only the observations without missing values. Second, we used the multiple imputation method to impute ten versions of complete dataset. The multiple imputation allows for the uncertainty about the missing data by creating several different plausible imputed data sets and appropriately combining results obtained from each of them. Models were separately developed for 10 complete datasets and the results were combined for the final inference [24].

## Results

### Baseline demographics and clinical characteristics

The clinical characteristics of both study cohorts are summarized in Table III and IV in the online-only supplements. In total, there were 265,897 patients (65.1% with stage 1 and 2 hypertension) in the age 60–65 cohort and 211,116 patients (76.3% with stage 1 and 2 hypertension) in the age 70–75 cohort. The proportion of patients with at least 2 BP measurements within a 2 year period was 58.4% of in the age 60–65 cohort and 64.4% in the age 70–75 cohort. For the age 60–65 cohort, 46.7% were male and 2.5% and 6.7% had a history of stroke and diabetes, respectively; for the age 70–75 cohort, 43% were male and 2.8 and 7.7% had a history of stroke and diabetes, respectively.

### Incidence rate of vascular dementia

**Fig. 1:**
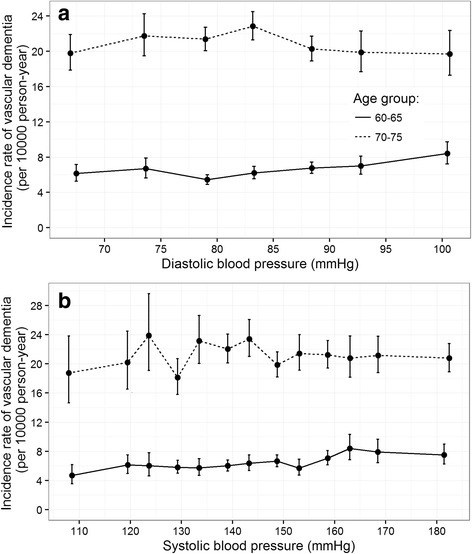
Age and sex-standardized rate of vascular dementia per 10,000 person-years by (**a**) diastolic and (**b**) systolic blood pressure. Blood pressure were categorized based on the 5 mmHg interval

We explored the association of systolic and diastolic blood pressure with vascular dementia by estimating the age and sex standardized incidence rate of vascular dementia for each category of SBP and DBP (Fig. 1). There was no clear trend between VaD and systolic and diastolic blood pressure in the age 70–75 cohort. In the age 60–65 cohort, the incidence rate of VaD for individuals with systolic blood pressures below 140 mmHg was 5.8 per 10,000 person-years, which was lower than individuals with systolic blood pressures above 160 mmHg (7.8 per 10,000 person-years).

### Age 60–65 cohort

The proportion of male patients decreased with higher BP levels (Table III). The proportion of current smokers decreased with higher BP levels while the proportion of individuals with obesity increased. Individuals with normal and stage 2 hypertension have slightly more missing values than the other two groups. The proportion of patients with a history of diabetes, stroke and TIA increased at higher BP levels while the proportion of patients with a history of depression, head injury, and Parkinson’ disease decreased at higher BP levels. Around 15% to 22% of individuals died, with the highest proportion of death for those with stage 2 hypertension. The mean follow up time was 11.0 years (standard deviation (SD): 4.97).

**Fig. 2:**
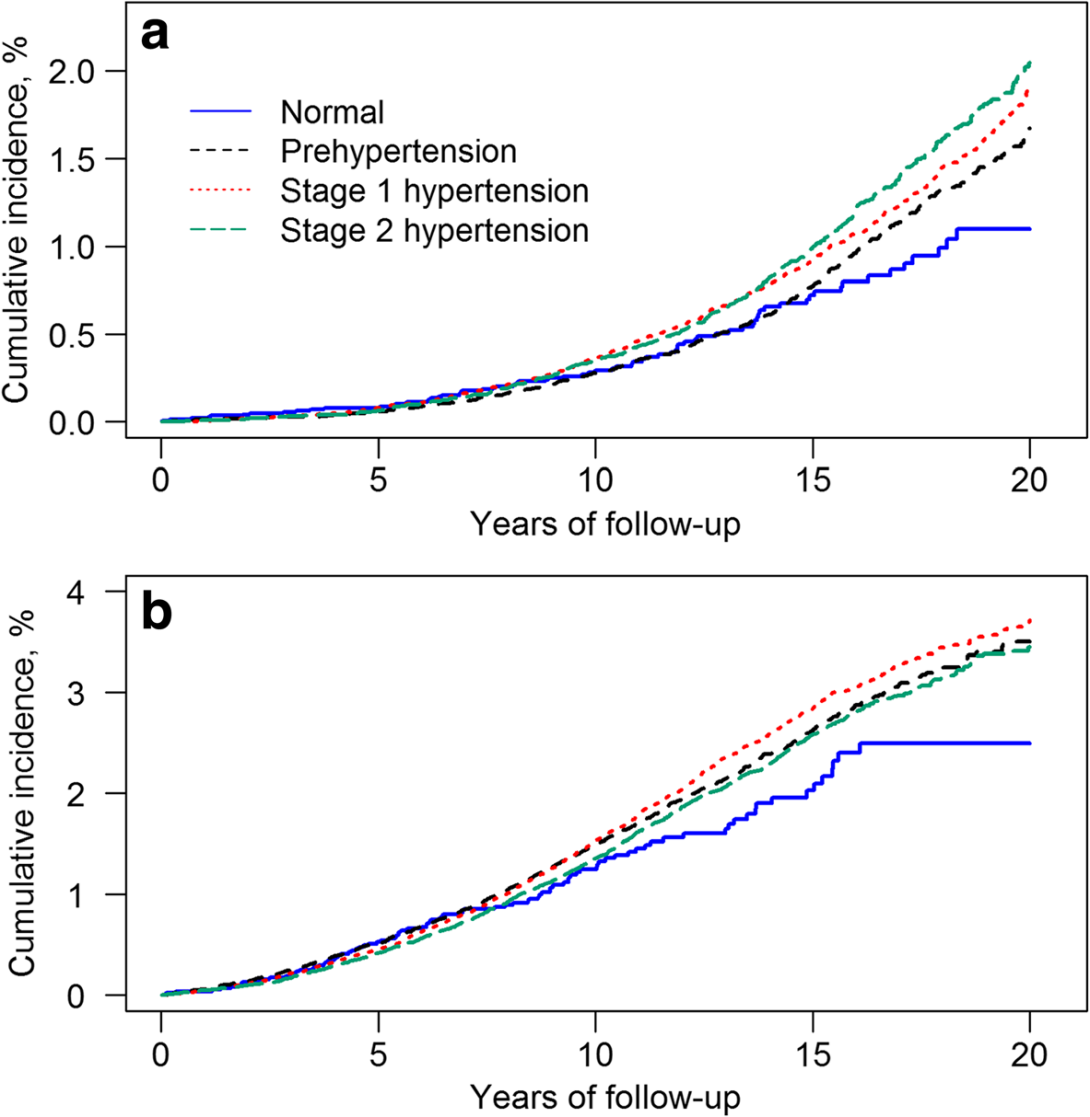
Comparison of the cumulative incidence of vascular dementia for individuals with normal, prehypertension, stage 1 and 2 hypertension in the age 60–65 (**a**) and age 70-75cohorts (**b**), adjusting for the competing risk of death

Figure 2 (a) shows the cumulative incidence of VaD for the age 60–65 cohort after accounting for the competing risk of death. At 20 years of follow-up, the cumulative incidence of VaD in patients with stage 2 hypertension was approximately 2% while the cumulative incidence of VaD in patients with normal blood pressure was 1%. The difference in the cumulative incidence of VaD for patients with different BP levels rose after the 10 years of follow-up. For the untreated group without use of antihypertensive drugs, after adjusting for age and sex, the risk of VaD increased with higher BP levels, with a hazard ratio (HR) of 1.50 (95% confidence interval: 1.02, 2.20) for prehypertension, 1.84 (1.26, 2.68) for stage 1 hypertension, and 2.09 (1.41, 3.10) for stage 2 hypertension (Table 1). The increased risk of VaD with increasing of BP levels remained significant after adjusting for potential confounding factors (age, sex, BMI, smoking status, and history of diabetes, stroke, head injury, depression and Parkinson’s disease). After multiple imputation, the HRs for VaD increased slightly in each BP levels compared to age and sex adjusted HRs. For the treated group, there was no increasing risk of VaD with increasing of BP levels. For each BP level, our data showed that the treated group had higher crude incidence of VaD than untreated group (Table 1). PP levels were not identified as a risk predictor for VaD in both untreated and treated group (Table 2). This pattern was consistent after adjusting the other confounding factors and imputing missing values (Table 2). However, after stratified by BP, PP was still not associated with VaD except for the untreated group with stage 1 hypertension (Table 3). Hazard ratios for the two groups with PP > 60 mmHg were inconsistent before and after imputing missing value. Additional file 1: Table S3 indicated that individuals with normal and stage 2 hypertension had more missing values in smoking status and BMI categories than the other two groups. Our further analyses on untreated patients with stage 1 hypertension showed that the individuals with PP levels of ≤50 mmHg and PP > 70 mmHg had more missing values than the other two groups.

**Table 1:** Association between blood pressure levels and vascular dementia in age 60–65 and 70–75 cohorts

Blood pressure levels	*N*	Death, %	VaD, %	Hazard ratio^a^ (95% CI)	*P* value	Hazard ratio^b^ (95% CI)	*P* value	Hazard ratio^c^ (95% CI)	*P* value
Age 60–65 cohort – untreated with antihypertensive drug									
Normal	10,335	13.49	0.39	1.00 (reference)				1.00 (reference)	
Prehypertension	56,231	13.24	0.55	1.50 (1.02, 2.20)	0.04	1.25 (0.88, 1.77)	0.21	1.53 (1.04, 2.25)	0.03
Stage 1 hypertension	71,191	15.73	0.66	1.84 (1.26, 2.68)	0.002	1.46 (1.03, 2.05)	0.032	1.90 (1.30, 2.78)	0.001
Stage 2 hypertension	29,197	20.86	0.83	2.09 (1.41, 3.10)	<0.001	1.49 (1.04, 2.13)	0.03	2.19 (1.48, 3.26)	< 0.001
Age 60–65 cohort – treated with antihypertensive drug									
Normal	3344	25.60	0.84	1.00 (reference)		1.00 (reference)		1.00 (reference)	
Prehypertension	22,707	18.64	0.66	0.92 (0.56, 1.52)	0.75	0.90 (0.58, 1.40)	0.64	0.91 (0.55, 1.50)	0.71
Stage 1 hypertension	47,227	17.50	0.75	1.05 (0.65, 1.69)	0.84	0.97 (0.63, 1.48)	0.88	1.04 (0.65, 1.68)	0.86
Stage 2 hypertension	25,665	22.70	0.86	1.07 (0.65, 1.75)	0.79	0.92 (0.59, 1.42)	0.70	1.08 (0.66, 1.77)	0.76
Age 70–75 cohort – untreated with antihypertensive drug									
Normal	3269	32.82	1.50	1.00 (reference)		1.00 (reference)		1.00 (reference)	
Prehypertension	24,080	30.32	1.65	1.18 (0.82, 1.72)	0.37	1.07 (0.76, 1.52)	0.69	1.19 (0.82, 1.72)	0.37
Stage 1 hypertension	46,194	31.58	1.72	1.18 (0.82, 1.69)	0.38	1.13 (0.81, 1.59)	0.47	1.18 (0.82, 1.70)	0.37
Stage 2 hypertension	30,874	36.97	1.72	1.12 (0.77, 1.62)	0.55	1.09 (0.77, 1.53)	0.64	1.14 (0.79, 1.65)	0.49
Age 70–75 cohort – treated with antihypertensive drug									
Normal	2854	46.74	1.51	1.00 (reference)		1.00 (reference)		1.00 (reference)	
Prehypertension	20,032	36.74	1.81	1.49 (0.94, 2.35)	0.09	1.10 (0.78, 1.54)	0.60	1.50 (0.95, 2.37)	0.085
Stage 1 hypertension	48,593	33.30	1.91	1.76 (1.12, 2.74)	0.013	1.17 (0.84, 1.63)	0.37	1.78 (1.14, 2.79)	0.011
Stage 2 hypertension	36,220	38.65	1.89	1.56 (1.00, 2.45)	0.052	1.11 (0.79, 1.54)	0.56	1.60 (1.02, 2.51)	0.041

*VaD* Vascular dementia, *CI* Confidence interval

^a^Adjusted for sex (female = 1) and age

^b^Adjusted for sex (female = 1), age, body mass index, smoking status, history of diabetes, stroke, transient ischemic attack, head injury and Parkinson’ disease while excluding any individuals with missing values

^c^Adjusted for sex (female = 1), age, body mass index, smoking status, history of diabetes, stroke, transient ischemic attack, head injury and Parkinson’ disease with missing values imputed by multiple imputation method

**Table 2:** Association between pulse pressure levels and vascular dementia in age 60–65 and 70–75 cohorts

Pulse pressure (PP) (mmHg)	*N*	Death, %	VaD, %	Hazard ratio^a^ (95% CI)	*P* value	Hazard ratio^b^ (95% CI)	*P* value	Hazard ratio^c^ (95% CI)	*P* value
Age 60–65 cohort – untreated with antihypertensive drug									
PP ≤ 50	51,721	14.17	0.55	1.00 (reference)		1.00 (reference)		1.00 (reference)	
50 < PP ≤ 60	49,975	14.49	0.64	1.11 (0.93, 1.33)	0.24	1.72 (0.98, 1.40)	0.77	1.11 (0.93, 1.34)	0.24
60 < PP ≤ 70	37,159	16.02	0.68	1.18 (0.97, 1.43)	0.10	1.19 (0.98, 1.44)	0.74	1.18 (0.97, 1.43)	0.1
PP > 70	28,099	19.97	0.74	1.15 (0.93, 1.42)	0.19	1.19 (0.97, 1.45)	0.89	1.15 (0.94, 1.42)	0.18
Age 60–65 cohort – treated with antihypertensive drug									
PP ≤ 50	15,556	21.43	0.71	1.00 (reference)		1.00 (reference)		1.00 (reference)	
50 < PP ≤ 60	26,748	17.83	0.73	1.03 (0.78, 1.35)	0.86	1.03 (0.81, 1.33)	0.80	1.02 (0.77, 1.33)	0.91
60 < PP ≤ 70	28,574	17.66	0.79	1.15 (0.88, 1.49)	0.31	1.11 (0.87, 1.41)	0.41	1.13 (0.87, 1.47)	0.36
PP > 70	28,065	21.48	0.78	1.03 (0.79, 1.36)	0.81	0.98 (0.76, 1.26)	0.87	1.02 (0.78, 1.34)	0.88
Age 70–75 cohort – untreated with antihypertensive drug									
PP ≤ 50	18,081	32.91	1.54	1.00 (reference)		1.00 (reference)		1.00 (reference)	
50 < PP ≤ 60	24,951	31.28	1.64	1.06 (0.88, 1.28)	0.51	1.12 (0.93, 1.34)	0.23	1.06 (0.88, 1.27)	0.57
60 < PP ≤ 70	27,114	31.78	1.74	1.08 (0.90, 1.29)	0.43	1.21 (1.12, 1.44)	0.031	1.07 (0.89, 1.28)	0.48
PP > 70	34,271	35.02	1.79	1.08 (0.91, 1.29)	0.38	1.15 (0.94, 1.37)	0.10	1.08 (0.91, 1.29)	0.37
Age 70–75 cohort – treated with antihypertensive drug									
PP ≤ 50	10,268	42.75	1.76	1.00 (reference)		1.00 (reference)		1.00 (reference)	
50 < PP ≤ 60	20,576	35.92	1.82	1.27 (1.00, 1.61)	0.046	1.00 (0.82, 1.22)	0.98	1.27 (1.00, 1.60)	0.05
60 < PP ≤ 70	29,672	33.86	1.96	1.39 (1.11, 1.73)	0.004	1.07 (0.89, 1.28)	0.50	1.38 (1.10, 1.73)	0.005
PP > 70	47,183	36.13	1.86	1.17 (0.94, 1.46)	0.15	0.97 (0.81, 1.16)	0.73	1.18 (0.94, 1.46)	0.15

VaD: Vascular dementia; CI: Confidence interval

^a^Adjusted for sex (female = 1) and age

^b^Adjusted for sex (female = 1), age, body mass index, smoking status, history of diabetes, stroke, transient ischemic attack, head injury and Parkinson’ disease while excluding any individuals with missing values

^c^Adjusted for sex (female = 1), age, body mass index, smoking status, history of diabetes, stroke, transient ischemic attack, head injury and Parkinson’ disease with missing values imputed by multiple imputation method

**Table 3:** Association between Pulse pressure levels and vascular dementia stratified by blood pressure levels in age 60–65 cohort

Pulse pressure (PP) (mmHg)	*N*	Death, %	VaD, %	Hazard ratio^a^ (95% CI)	*P* value	Hazard ratio^b^ (95% CI)	*P* value	Hazard ratio^c^ (95% CI)	*P* value
Age 60–65 cohort – untreated with antihypertensive drug									
Prehypertension									
PP ≤ 50	32,434	13.56	0.52	1.00 (reference)		1.00 (reference)		1.00 (reference)	
50 < PP ≤ 60	20,007	12.60	0.59	1.01 (0.77, 1.33)	0.95	1.14 (0.88, 1.48)	0.32	0.99 (0.75, 1.30)	0.93
60 < PP ≤ 70	3503	13.79	0.51	0.92 (0.53, 1.60)	0.77	1.07 (0.64, 1.80)	0.79	0.88 (0.51, 1.53)	0.65
PP > 70	217	15.21	0.92	2.15 (0.53, 8.71)	0.28	2.63 (0.64, 1.08)	0.18	2.00 (0.49, 8.15)	0.33
Stage 1 hypertension									
PP ≤ 50	8330	16.47	0.79	1.00 (reference)		1.00 (reference)		1.00 (reference)	
50 < PP ≤ 60	27,993	15.63	0.68	0.79 (0.58, 1.07)	0.13	1.02 (0.74, 1.40)	0.93	0.78 (0.57, 1.06)	0.11
60 < PP ≤ 70	26,983	15.28	0.65	0.70 (0.51, 0.96)	0.028	0.97 (0.70, 1.34)	0.84	0.68 (0.49, 0.94)	0.018
PP > 70	7885	16.89	0.55	0.59 (0.38, 0.91)	0.016	0.95 (0.62, 1.44)	0.80	0.56 (0.36, 0.87)	0.009
Stage 2 hypertension									
PP ≤ 50	1162	19.88	0.86	1.00 (reference)		1.00 (reference)		1.00 (reference)	
50 < PP ≤ 60	1393	19.45	0.72	1.10 (0.38, 3.15)	0.86	0.62 (0.23, 1.67)	0.35	1.09 (0.38, 3.14)	0.87
60 < PP ≤ 70	6647	20.19	0.92	1.41 (0.61, 3.28)	0.42	0.85 (0.42, 1.73)	0.65	1.42 (0.61, 3.29)	0.42
PP > 70	19,995	21.24	0.81	1.02 (0.45, 2.31)	0.96	0.80 (0.41, 1.57)	0.52	1.03 (0.46, 2.34)	0.94
Age 60–65 cohort – treated with antihypertensive drug									
Prehypertension									
PP ≤ 50	9363	20.06	0.68	1.00 (reference)		1.00 (reference)		1.00 (reference)	
50 < PP ≤ 60	11,113	17.39	0.62	0.84 (0.57, 1.23)	0.37	0.97 (0.68, 1.39)	0.87	0.83 (0.56, 1.22)	0.33
60 < PP ≤ 70	2131	19.05	0.80	1.19 (0.66, 2.15)	0.57	1.41 (0.81, 2.47)	0.23	1.13 (0.61, 2.10)	0.70
PP > 70	100	17.00	0.00	N/A	N/A	N/A	N/A	N/A	N/A
Stage 1 hypertension									
PP ≤ 50	2648	20.70	0.72	1.00 (reference)		1.00 (reference)		1.00 (reference)	
50 < PP ≤ 60	14,601	17.81	0.78	1.39 (0.74, 2.62)	0.30	1.11 (0.65, 1.88)	0.71	1.38 (0.73, 2.59)	0.32
60 < PP ≤ 70	21,800	16.64	0.75	1.32 (0.71, 2.47)	0.38	1.08 (0.64, 1.81)	0.78	1.29 (0.69, 2.40)	0.43
PP > 70	8178	18.22	0.69	1.31 (0.67, 2.55)	0.43	1.01 (0.57, 1.79)	0.97	1.24 (0.63, 2.42)	0.53
Stage 2 hypertension									
PP ≤ 50	405	26.67	0.49	1.00 (reference)		1.00 (reference)		1.00 (reference)	
50 < PP ≤ 60	835	21.92	1.08	1.68 (0.35, 8.14)	0.52	1.78 (0.37, 8.50)	0.47	1.61 (0.33, 7.81)	0.56
60 < PP ≤ 70	4638	21.84	1.01	1.46 (0.35, 6.17)	0.60	1.49 (0.35, 6.26)	0.59	1.41 (0.33, 5.93)	0.64
PP > 70	19,787	22.85	0.83	1.09 (0.26, 4.51)	0.90	1.29 (0.31, 5.33)	0.73	1.02 (0.25, 4.24)	0.98

VaD: Vascular dementia; CI: Confidence interval

^a^Adjusted for sex (female = 1) and age

^b^Adjusted for sex (female = 1), age, body mass index, smoking status, history of diabetes, stroke, transient ischemic attack, head injury and Parkinson’ disease while excluding any individuals with missing values

^c^Adjusted for sex (female = 1), age, body mass index, smoking status, history of diabetes, stroke, transient ischemic attack, head injury and Parkinson’ disease with missing values imputed by multiple imputation method

### Age 70–75 cohort

The proportion of male patients decreased with higher BP levels, ranging from 55.2 to 37.1% (Table IV). Similar to the late midlife cohort, the proportion of patients with obesity increased at higher BP levels and the proportion of patients with a history of depression, head injury, and Parkinson’s disease decreased at higher BP levels. In contrast to the age 60–65 cohort, the proportion of patients with a history of stroke, TIA, and diabetes decreased at higher BP levels. During follow-up, 32 to 39% of individuals died, with highest proportion of death in those with stage 1 hypertension. The mean follow-up time was 8.84 years (SD: 4.55).

Figure 2 (b) shows the cumulative incidence curves of VaD for the late life cohort after accounting for the competing risk of death. At 20 years of follow up, the cumulative incidence of VaD in patients with stage 1 and 2 hypertension was 3.3%, while the cumulative incidence of VaD in patients with normal blood pressure was 2.4%. The difference in the cumulative incidence of VaD for patients with different BP levels rose after 10 years of follow-up. Similar with the 60–65 cohort, the individuals in treated hypertension group had higher incidence of VaD than those in untreated group (Table 1). For the untreated group, there was no statistically significant association between the risk of VaD and BP levels (Table 1). For the treated group, the association was observed between BP and VaD for stage 1 and 2 hypertension patients after adjusting for potential confounding factors and with the imputed missing values. This association was no longer significant after excluding individuals with any missing values. Similar results were observed for analysis on PP levels (Table 2). The analyses stratified by BP levels indicated that PP level is not an independent risk factor for VaD (Table 4). Similar with the cohort aged 60–65 years, there are more missing values in smoking status and BMI categories in individuals with normal and stage 2 hypertension than the other two groups (Additional file 1: Table S4).

**Table 4:** Association between pulse pressure levels and vascular dementia stratified by blood pressure levels in age 70–75 cohort

Pulse pressure (PP) (mmHg)	*N*	Death, %	VaD, %	Hazard ratio^a^ (95% CI)	*P* value	Hazard ratio^b^ (95% CI)	*P* value	Hazard ratio^c^ (95% CI)	*P* value
Age 70–75 cohort – untreated with antihypertensive drug									
Prehypertension									
PP ≤ 50	11,244	31.56	1.51	1.00 (reference)		1.00 (reference)		1.00 (reference)	
50 < PP ≤ 60	9872	29.55	1.81	1.12 (0.86, 1.45)	0.41	1.20 (0.93, 1.54)	0.16	1.09 (0.84, 1.41)	0.54
60 < PP ≤ 70	2669	27.69	1.72	1.34 (0.93, 1.93)	0.12	1.20 (0.83, 1.73)	0.34	1.30 (0.90, 1.88)	0.16
70 < PP	295	32.54	1.02	0.60 (0.15, 2.44)	0.48	0.61 (0.15, 2.46)	0.49	0.59 (0.15, 2.40)	0.46
Stage 1 hypertension									
PP ≤ 50	3312	36.75	1.72	1.00 (reference)		1.00 (reference)		1.00 (reference)	
50 < PP ≤ 60	14,051	32.02	1.54	0.91 (0.64, 1.30)	0.61	1.01 (0.71, 1.44)	0.95	0.90 (0.63, 1.28)	0.55
60 < PP ≤ 70	19,583	30.94	1.79	0.97 (0.69, 1.37)	0.85	1.21 (0.86, 1.70)	0.28	0.95 (0.67, 1.34)	0.76
70 < PP	9248	30.43	1.83	0.99 (0.68, 1.44)	0.97	1.13 (0.78, 1.64)	0.52	0.97 (0.67, 1.41)	0.88
Stage 2 hypertension									
PP ≤ 50	556	38.85	0.90	1.00 (reference)		1.00 (reference)		1.00 (reference)	
50 < PP ≤ 60	757	39.37	1.32	1.95 (0.52, 7.34)	0.32	1.25 (0.36, 4.28)	0.72	1.94 (0.52, 7.32)	0.33
60 < PP ≤ 70	4836	37.39	1.53	1.64 (0.51, 5.27)	0.41	1.40 (0.51, 3.86)	0.52	1.67 (0.52, 5.36)	0.39
70 < PP	24,725	36.77	1.78	2.08 (0.67, 6.47)	0.21	1.59 (0.59, 4.27)	0.36	2.10 (0.68, 6.53)	0.20
Age 70–75 cohort – treated with antihypertensive drug									
Prehypertension									
PP ≤ 50	5994	40.19	1.84	1.00 (reference)		1.00 (reference)		1.00 (reference)	
50 < PP ≤ 60	9948	35.27	1.78	1.27 (0.92, 1.76)	0.14	0.95 (0.73, 1.24)	0.71	1.23 (0.89, 1.70)	0.21
60 < PP ≤ 70	3728	34.74	1.88	1.38 (0.94, 2.05)	0.1	1.02 (0.73, 1.42)	0.91	1.30 (0.88, 1.93)	0.18
70 < PP	362	40.33	1.38	0.87 (0.27, 2.79)	0.81	0.81 (0.32, 2.01)	0.64	0.80 (0.25, 2.58)	0.71
Stage 1 hypertension									
PP ≤ 50	1511	44.08	1.99	1.00 (reference)		1.00 (reference)		1.00 (reference)	
50 < PP ≤ 60	9789	36.02	1.87	0.88 (0.56, 1.38)	0.57	0.82 (0.54, 1.23)	0.33	0.87 (0.55, 1.36)	0.53
60 < PP ≤ 70	22,333	32.49	2.02	0.92 (0.60, 1.42)	0.71	0.84 (0.57, 1.24)	0.39	0.90 (0.59, 1.39)	0.64
70 < PP	14,960	31.66	1.75	0.70 (0.45, 1.09)	0.12	0.71 (0.48, 1.06)	0.10	0.68 (0.44, 1.07)	0.096
Stage 2 hypertension									
PP ≤ 50	291	47.08	1.72	1.00 (reference)		1.00 (reference)		1.00 (reference)	
50 < PP ≤ 60	480	43.96	1.67	0.76 (0.17, 3.39)	0.72	1.17 (0.30, 4.54)	0.82	0.77 (0.17, 3.42)	0.73
60 < PP ≤ 70	3588	41.44	1.67	0.90 (0.28, 2.91)	0.86	1.01 (0.31, 3.23)	0.99	0.90 (0.28, 2.92)	0.86
70 < PP	31,861	38.18	1.92	1.04 (0.34, 3.24)	0.94	1.12 (0.36, 3.48)	0.85	1.05 (0.34, 3.25)	0.94

*VaD* Vascular dementia, *CI* Confidence interval

^a^Adjusted for sex (female = 1) and age

^b^Adjusted for sex (female = 1), age, body mass index, smoking status, history of diabetes, stroke, transient ischemic attack, head injury and Parkinson’ disease while excluding any individuals with missing values

^c^Adjusted for sex (female = 1), age, body mass index, smoking status, history of diabetes, stroke, transient ischemic attack, head injury and Parkinson’ disease with missing values imputed by multiple imputation method

## Discussion

In this study, there is an increasing risk of VaD with increasing levels in the age 60–65 cohort without antihypertensive drug, while such an association was not observed in the age 70–75 cohort. Therefore, optimal control of late midlife BP levels and prescription of antihypertensive drugs may be important to reduce the risk of VaD. Further research is still needed to clarify the role of late-life high blood pressure on the prognosis of VaD, especially for patients aged 70 and above.

Several studies that have previously examined the association between VaD and midlife BP levels reached similar conclusions. The Hisayama study found that subjects with prehypertension, stage 1, and stage 2 hypertension in midlife (around 58 years) had higher risk of VaD after adjusting for potential risk factors [5]. The Honolulu Asia Aging Study recommended lowering midlife (aged 45–68 years) systolic blood pressure as a public health strategy to reduce late-life dementia after demonstrating high of risk of VaD for patients with untreated hypertension [8]. High blood pressure at middle age implies a long-term cumulative effect, which leads to increased severity of atherosclerosis and more vascular comorbidities in late life [7]. Patients with high blood pressure in midlife were at increased risk of VaD, regardless of late-life BP levels, [5] likely as midlife hypertension leads to damage that becomes irreversible in the late life. Our findings highlight the potential importance of blood pressure control in the midlife period.

Trials of antihypertensive therapy have not yet clarified the impact of blood pressure lowering on vascular dementia. Our study identified the increasing risk of VaD with an increase of BP levels for the individuals without the use of antihypertensive drugs, while there was no association between BP levels and VaD in the treated individuals. A recent meta-analysis of trials and cohort studies showed promising findings that diuretic-based lowering of blood pressure prevents dementia [25]. Other trials and a meta-analysis of trials have produced inconsistent results, likely related to the trials being retrospectively designed, with weaker assessments of dementia and its components, and follow-up times that are too short [26–28]. Additional randomized controlled trials with more sensitive and specific tests for dementia and its components, as well as longer follow-up periods, may be needed to clarify the impact of hypertension on dementia. However, given the strong evidence that antihypertensive therapy has beneficial effects on cardiovascular outcomes, most trial designs would be unethical to conduct. The recent SPRINT trial examining newer blood pressure targets is an exception and its results on dementia are anticipated [29].

Increases of PP in midlife is closely related to the increases of SBP. The associations between PP and VaD were not significant when stratified analyses at each BP level and treatment status were conducted. Therefore, PP was not independently associated with VaD. This is consistent with results from the other study [30]. Pulse pressure may not be an adequate measure of arterial stiffness and pulsatile flow as pulse wave velocity has been shown to prevent cognitive decline better than measures of blood pressure [31].

After adjusting the competing risk of death and other risk factors, our study found no association between BP levels and risk of vascular dementia in the age 70–75 cohort. This could be due to several reasons. First, there is a graded increase in the risk of stroke with advancing age, [32] with a doubling in the stroke rate with each successive 10 years after age 55 [33, 34]. Second, after acute stroke, patients with high blood pressure levels have higher mortality rate [35]. As a result, survivors of strokes are more likely to have normal blood pressure (as shown in Table II). Third, a decline of blood pressure happens in the pathogenesis of dementia [36]. This could weaken the association between high blood pressure levels and risk of vascular dementia in late-life. Fourth, competing risk of death is more significant in later life. There is a potential “floor effect” of death on the development of vascular dementia. In our study, 40% of patients in the late-life cohort died during follow-up. The patients were likely to die before the development and diagnosis of vascular dementia. To assess the true effects of vascular risk factor on the development of vascular dementia, it may be necessary to use brain autopsy and neuroimaging to identify dementia cases.

Clear association of late midlife high BP and VaD in untreated group supports the need to treat and control BPs for patients with late midlife hypertension. According to health surveys, around half of patients with hypertension do not have their blood pressure under control [37]. In our study, only 20 to 25% patients with stage 1 and 2 hypertension had a diagnosis of hypertension in EMR record. Patients with a code of hypertension diagnosis in EMR are more likely to receive treatments [38, 39]. Therefore, greater effort must be made for the early diagnosis and treatment of hypertension, especially in midlife.

Our study is a large, population-based cohort study, which enabled us to examine the association of vascular dementia related to late midlife and late life blood pressure. We also accounted for the competing risk of death and adjusted for several important risk factors for dementia. However, several limitations should be noted. First, selection bias might exist since we only included patients who had at least one blood pressure measurement before the ages of 65 and 75. Around 40% of patients in the study cohorts had only a single measurement of blood pressure and this would have led to some degree of misclassification of blood pressure levels. Also, patients with hypertension requiring frequent visits may increase the reporting of dementia. Second, there are potential problems with misclassification or misdiagnosis of vascular dementia. This issue stems from difficulty with the clinical differentiation and classification of dementia subtypes; [40] there is inconsistency in applying commonly used classification systems for the dementia diagnoses. However, the diagnosis in EMR is closely relevant to routine clinical care and has shown good agreement with physician diagnosis and other data sources. Third, people with higher levels of blood pressure may be more apt to receive antihypertensive treatment than those with lower levels, reversing the relationship of hypertension to stroke and dementia. To alleviate this issue, we stratified the analysis using the drug use status. Lastly, missing or incomplete information on some risk factors could impact the validity of results. Our analysis showed inconsistency of results with and without imputing missing values in some subcategories. Some potential confounders were not recorded well in the database and subsequently excluded from the analysis. For example, exercise levels were only recorded in 22% of individuals in the study cohorts and were not included in the analysis.

## Conclusions

In summary, our study indicates that high blood pressure between the ages of 60 and 65 is a significant risk factor for vascular dementia in an untreated late midlife cohort. Given the strength and consistency of this evidence, greater efforts should be made to diagnose hypertension early, and control blood pressure for hypertensive patients in the prevention to better prevent vascular dementia.

## Additional files

Additional files 1: List of Read codes (Table S1) and antihypertensive drugs (Table S2), Clinical characteristics of study cohort with the age of 60 to 65 (Table S3) and 70 to 75 (Table S4). (DOCX 36 kb)
