# Supplementary material for: Blood pressure at age 60–65 versus age 70–75 and vascular dementia: a population based observational study
Source: BMC Geriatr. 2017 Oct 27;17:252. doi: 10.1186/s12877-017-0649-3 (PMC5658926; doi:10.1186/s12877-017-0649-3)
Supplement: Supplementary file 1 — List of Read codes (Table S1) and antihypertensive drugs (Table S2), Clinical characteristics of study cohort with the age of 60 to 65 (Table S3) and 70 to 75 (Table S4). (DOCX 36 kb) [file 12877_2017_649_MOESM1_ESM.docx]

Table S1. List of Read codes

| Condition | Read code | Explanation |
| --- | --- | --- |
| Stroke | G61 | Intracerebral hemorrhage / CVA - cerebrovascular accid due to intracerebral hemorrhage / Stroke due to intracerebral hemorrhage |
|  | G63y | Other pre-cerebral artery occlusion |
|  | G64 | Cerebral arterial occlusion / CVA - cerebral artery occlusion / Infarction - cerebral / Stroke due to cerebral arterial occlusion |
|  | G66 | Stroke and cerebrovascular accident unspecified / CVA unspecified / Stroke unspecified / CVA - Cerebrovascular accident unspecified |
|  | G676 | Nonpyogenic venous sinus thrombosis |
|  | G6W | Cerebral infarct due unspocclus/stenos pre-cerebral arteries |
|  | G6X | Cerebral infarction due/unspocclus or stem/cerebral artrs |
|  | Gyu6 | Additional circulatory system disease classification terms |
| Diabetes | C10 | Diabetes mellitus |
| Depression | E112 | Single major depressive episode / Agitated depression / Endogenous depression first episode / Endogenous depression first episode / Endogenous depression |
|  | E113 | [Recurrent major depressive episode / Endogenous depression - recurrent](javascript:void(0);) |
|  | E118 | E118 - Seasonal affective disorder |
|  | E11y2 | Other and unspecified manic-depressive psychoses NOS |
|  | E11z2 | Masked depression |
|  | E130 | Reactive depressive psychosis / Psychotic reactive depression |
|  | E135 | Agitated depression |
|  | E2003 | Anxiety with depression |
|  | E291 | Prolonged depressive reaction |
|  | E2B | Depressive disorder NEC |
|  | Eu204 | Post-schizophrenic depression |
|  | Eu251 | Schizoaffective disorder, depressive type / Schizoaffective psychosis, depressive type / Schizophreniform psychosis, depressive type |
|  | Eu32 | Depressive episode / Single episode of depressive reaction /Single episode of psychogenic depression / Single episode of reactive depression |
| Parkinson’s disease | F12 | Parkinson's disease |
| Head injury | S0 | Fracture of skull |
|  | S6 | Intracranial injury excluding those with skull fracture |
| Transient Ischemic Attack (TIA) | G65 | G65 - Transient cerebral ischemia / Drop attack / Transient ischemic attack / Vertebro-basilar insufficiency |
|  | Fyu55 | Other transient cerebral ischemic attacks and related syndromes |

Table S2. List of antihypertensive drugs*.

| Drug name | Number of corresponding drug codes | Drug name | Number of corresponding drug codes |
| --- | --- | --- | --- |
| Acebutolol | 8 | Labetalol | 22 |
| Amiloride | 43 | Lisinopril | 43 |
| Amlodipine | 30 | Losartan | 16 |
| Atenolol | 65 | Methyldopa | 24 |
| Bendroflumethiazide | 46 | Metolazone | 4 |
| Bisoprolol | 41 | Metoprolol | 40 |
| Candesartan | 10 | Nadolol | 10 |
| Captopril | 48 | Nicardipine | 9 |
| Chlortalidone | 12 | Nifedipine | 108 |
| Cilazapril | 10 | Oxprenolol | 21 |
| Digitoxin | 2 | Pentaerythritol Tetranitrate | 4 |
| Digoxin | 34 | Perindopril | 23 |
| Diltiazem | 145 | Phentolamine | 2 |
| Doxazosin | 37 | Pindolol | 9 |
| Enalapril | 55 | Prazosin | 21 |
| Eprosartan | 6 | Propranolol | 88 |
| Etacrynic Acid | 1 | Quinapril | 11 |
| Felodipine | 36 | Ramipril | 73 |
| Fosinopril | 4 | Spironolactone | 55 |
| Furosemide | 76 | Telmisartan | 9 |
| Glyceryl Trinitrate | 51 | Terazosin | 17 |
| Hydrochlorothiazide | 75 | Timolol | 61 |
| Indapamide | 16 | Trandolapril | 13 |
| Irbesartan | 10 | Triamterene | 11 |
| Isosorbide Dinitrate | 19 | Valsartan | 24 |
| Isosorbide Mononitrate | 44 | Verapamil | 60 |

* The drug list combined all the drugs listed in Hemmelgarn et al. 2008 and Williamson et al. 2014.

Hemmelgarn BR, Chen G, Walker R, et al. Trends in antihypertensive drug prescriptions and physician visits in Canada between 1996 and 2006. Can J Cardiol 2008;24(6):507-512.

Williamson T, Green ME, Birtwhistle R, et al. Validating the 8 CPCSSN case definitions for chronic disease surveillance in a primary care database of electronic health records. Ann Fam Med 2014; 12(4): 367-372.

Table S3. Clinical characteristics of study cohort with the age of 60 to 65.

|  | Blood pressure levels defined by JNC-7 | | | |  |
| --- | --- | --- | --- | --- | --- |
| Characteristics | Normal  N=13679 | Prehypertension  N=78939 | Stage 1 hypertension  N=118418 | Stage 2 hypertension  N=54862 | P value |
| Male, % | 48.4 | 47.7 | 46.4 | 45.1 | <0.05 |
| Smoking status, % |  |  |  |  | <0.05 |
| Never smoked | 53.8 | 57.1 | 58.1 | 56.5 |  |
| Former smoker | 19.9 | 20.6 | 21.0 | 19.5 |  |
| Current smoker | 24.7 | 21.0 | 19.4 | 21.9 |  |
| Missing | 1.7 | 1.3 | 1.4 | 2.1 |  |
| BMI categories, % |  |  |  |  | <0.05 |
| Normal (< 25) | 43.3 | 31.9 | 23.9 | 20.9 |  |
| Overweight (25 to 30) | 30.3 | 35.8 | 37.0 | 34.8 |  |
| Obesity ( ≥ 30) | 9.0 | 15.9 | 22.6 | 26.3 |  |
| Missing | 17.4 | 16.5 | 16.5 | 18.1 |  |
| ≥2 times BP measurements, % | 44.9 | 53.3 | 61.7 | 61.7 | <0.05 |
| Diagnosis of hypertension,,% | 2.5 | 9.5 | 19.9 | 24.8 | <0.05 |
| History of diabetes, % | 4.4 | 6.5 | 7.1 | 6.7 | <0.05 |
| History of stroke, % | 1.1 | 1.4 | 1.5 | 1.5 | <0.05 |
| History of TIA, % | 1.5 | 1.5 | 1.6 | 1.7 | 0.05 |
| History of head injury, % | 1.4 | 1.3 | 1.1 | 0.9 | <0.05 |
| History of depression, % | 9.2 | 8.4 | 8.0 | 6.7 | <0.05 |
| History of PD, % | 0.6 | 0.3 | 0.2 | 0.2 | <0.05 |

JNC-7: The Seventh Report of the Joint National Committee on Prevention, Detection, Evaluation, and Treatment of High Blood Pressure; BMI: Body mass index; BP: blood pressure; TIA: Transient ischemic attack; PD: Parkinson’s disease; IQR: interquartile range.

Table S4. Clinical characteristics of study population with age of 70 to 75.

|  | Blood pressure levels defined by JNC-7 | | | |  |
| --- | --- | --- | --- | --- | --- |
| Characteristics | Normal  N=6123 | Prehypertension  N=44112 | Stage 1 hypertension  N=94787 | Stage 2 hypertension  N=67092 | P value |
| Male, % | 55.2 | 50 | 43.1 | 37.1 | <0.05 |
| Smoking status, % |  |  |  |  | <0.05 |
| Never smoked | 49.9 | 56.1 | 59.8 | 60.7 |  |
| Former smoker | 25.3 | 24.5 | 22.7 | 19.6 |  |
| Current smoker | 20.2 | 16.3 | 14.3 | 15.0 |  |
| Missing | 4.6 | 3.2 | 3.2 | 4.8 |  |
| BMI categories, % |  |  |  |  | <0.05 |
| Normal (< 25) | 41.5 | 33.6 | 28.3 | 26.1 |  |
| Overweight (25 to 30) | 27.1 | 32.6 | 33.9 | 31.6 |  |
| Obesity ( ≥ 30) | 8.3 | 13.0 | 16.3 | 16.9 |  |
| Missing | 23.1 | 20.8 | 21.5 | 25.4 |  |
| ≥2 times BP measurements, % | 51.7 | 61.1 | 66.8 | 64.4 | <0.05 |
| Diagnosis of hypertension,,% | 1.7 | 6.0 | 11.2 | 11.4 | <0.05 |
| History of diabetes, % | 6.7 | 8.8 | 7.9 | 6.7 | <0.05 |
| History of stroke, % | 3.3 | 3.0 | 2.9 | 2.6 | <0.05 |
| History of TIA, % | 4.1 | 3.8 | 3.8 | 3.3 | <0.05 |
| History of head injury, % | 1.8 | 1.4 | 1.3 | 1.0 | <0.05 |
| History of depression, % | 9.4 | 7.9 | 7.0 | 6.2 | <0.05 |
| History of PD, % | 2.0 | 1.0 | 0.7 | 0.4 | <0.05 |

JNC-7: The Seventh Report of the Joint National Committee on Prevention, Detection, Evaluation, and Treatment of High Blood Pressure; BMI: Body mass index; BP: blood pressure; TIA: Transient ischemic attack; PD: Parkinson’s disease; IQR: interquartile range.
